# Karyotype variation is indicative of subgenomic and ecotypic differentiation in switchgrass

**DOI:** 10.1186/1471-2229-12-117

**Published:** 2012-07-26

**Authors:** Hugh A Young, Gautam Sarath, Christian M Tobias

**Affiliations:** 1 Genomics and Gene Discovery Research Unit, USDA-Agricultural Research Service, Western Regional Research Center, 800 Buchanan Street, Albany, CA, 94710, USA; 2 USDA Central-East Regional Biomass Center, 137 Keim Hall, East Campus, UNL, Lincoln, NE, 68583, USA

**Keywords:** Bioenergy, Switchgrass, Cytogenetics, CHIAS IV, Perennial biofeedstocks, Dihaploids, Renewable energy, Polyploidy

## Abstract

**Background:**

Karyotypes can provide information about taxonomic relationships, genetic aberrations, and the evolutionary origins of species. However, differentiation of the tiny chromosomes of switchgrass (*Panicum virgatum* L.) and creation of a standard karyotype for this bioenergy crop has not been accomplished due to lack of distinguishing features and polyploidy.

**Results:**

A cytogenetic study was conducted on a dihaploid individual (2*n* = 2*X* = 18) of switchgrass to establish a chromosome karyotype. Size differences, condensation patterns, and arm-length ratios were used as identifying features and fluorescence in-situ hybridization (FISH) assigned 5S and 45S rDNA loci to chromosomes 7 and 2 respectively. Both a maize CentC and a native switchgrass centromeric repeat (PviCentC) that shared 73% sequence identity demonstrated a strong signal on chromosome 3. However, only the PviCentC probe labeled the centromeres of all chromosomes. Unexpected PviCentC and 5S rDNA hybidization patterns were consistent with severe reduction or total deletion of these repeats in one subgenome. These patterns were maintained in tetraploid and octoploid individuals. The 45S rDNA repeat produced the expected number of loci in dihaploid, tetraploid and octoploid individuals. Differences observed at the 5S rDNA loci between the upland and lowland ecotypes of switchgrass provided a basis for distinguishing these subpopulations.

**Conclusion:**

Collectively, these results provide a quantitative karyotype of switchgrass chromosomes. FISH analyses indicate genetic divergence between subgenomes and allow for the classification of switchgrass plants belonging to divergent genetic pools. Furthermore, the karyotype structure and cytogenetic analysis of switchgrass provides a framework for future genetic and genomic studies.

## Background

Polyploidy, the heritable duplication of whole genomes, is a key feature of plant diversification and is found in most, if not all, plant taxa [1]. Polyploidization can lead to speciation or creation of distinct reproductively-isolated cytotypes within a species. An ancient genome-doubling event and subsequent gene loss has shaped the genomes of all grass species [2]. Though an estimated 30-80% of angiosperms may be polyploid [3], the relative frequencies of allopolyploids which arise through hybridization of different species versus autopolyploids which arise within a single species are difficult to ascertain. Evolutionary processes that mask the origins of polyploid evolution such as introgression, deletion, concerted evolution, and mutation may create uncertainty. There is possible recurrent hybridization of genomes over time and interracial or close interspecific hybridization of genomes may produce similar outcomes. Incongruent tree-topologies from independent marker sets such as chloroplast and nuclear markers can suggest the presence of reticulate relationships, but these are not conclusive [4].

The interspecific relationships of taxa in the subgenus *Panicum* sensu stricto (s.s.) that comprises approximately 100 C4 grass species, including *Panicum virgatum* L. (switchgrass), haven’t yet been elucidated and likely represent a complex situation [5]. Because switchgrass is being pursued as a promising feedstock for renewable energy production in the United States and elsewhere, these relationships are of importance for understanding the breadth of germplasm that might be useful in a breeding program. The plant’s broad geographic range, water- and nutrient-use efficiency, and perennial growth habit make it ideal as a bioenergy crop [6-8]. The species encompasses multiple cytotypes with a basic chromosome number of nine [9,10], and a range of ploidy levels from diploid (2x) to duodecaploid (12x) [11-14]. As a polyploid species, switchgrass exhibits two basic subgenomes that are genetically divergent but maintain complete or near complete disomic inheritance [15]. Two separate ecotypes are distinguished cytologically and geographically [16]. Lowland accessions dominate the southern portion of the species range and are mainly tetraploid, while the upland accessions are usually either tetraploid or octoploid and dominate northern latitudes [17]. Extensive aneuploidy has also been described, especially within populations of octoploids, but these are likely present in all populations at varying levels [18].

Although artificial crosses between switchgrass cytotypes have been largely unsuccessful [19,20], questions still remain regarding historical hybridization between cytotypes, between upland and lowland ecotypes, and between several closely related species in the *Panicum* subgenus. These species may represent a common gene pool that has undergone repeated hybridization during “secondary contacts” of once isolated populations [21]. A recent analysis of switchgrass collections has demonstrated that there are two distinct centers of genetic diversity for lowland accessions represented by the Southern Great Plains and Eastern Gulf Coast while upland accessions appear genetically as one broadly distributed tetraploid and two octoploid lineages [22]. Using chloroplast sequence polymorphisms, molecular clock estimates have indicated that lowland and upland accessions diverged as early as 1.3 million years ago, but have possibly diverged on several occasions during recent cycles of glaciation [23,24].

In light of these uncertainties, independent methods to characterize genome structure would be useful for effective evaluation and utilization of germplasm resources. Cytogenetic analysis using in situ hybridization techniques have proven very useful in resolving genome constitution in polyploids and is an important tool in chromosome karyotyping [25]. In polyploid plants with small and highly similar chromosomes, karyotyping is aided by fluorescence in situ hybridization (FISH) using labeled total genomic DNA, repetitive sequences, or single copy probes. In particular, variation found at rDNA loci (45S and 5S rDNA) can sometimes be used to differentiate subgenomes or to distinguish between ecotypes of a species [26,27]. Chromosome reduction, breakage, or fusion during or after polyploidization can result in a gain or loss of these tandem repeat sequences. In the Triticeae, for example, both the location and order of rDNA loci differ extensively among related species [28]. FISH analyses using repetitive probes can further enable chromosome identification, and have been successfully employed in maize [29], rice [30], sugarcane [31], soybean [32], and pine [33].

In complex polyploid organisms such as switchgrass, the development of genotypes with reduced chromosome numbers would prove useful for breeding and genetic research [34]. Haploid plants, whether derived from a diploid or a polyploid, have *half* the chromosome number of the euploid form. Therefore, “haploid” plants derived from switchgrass tetraploids will have two copies of the basic chromosome number of nine (2*n* = 2*X* = 18). With a true haploid number of nine, the term “dihaploid” has been used to describe androgenic switchgrass lines containing two sets of homologous chromosomes [35] as well as gynogenic lines containing two sets of non-pairing homoeologous chromosomes [36]. The “dihaploid” terminology as well as the utility of such plant lines has been previously described for potato, barley, wheat, and several other species [37-39].

Early cytological studies in switchgrass focused mainly on overall counts of chromosomes rather than their individual morphology and molecular structure [40]. Our purpose here is to describe a quantitative karyotype of *P. virgatum* L. To simplify analysis, we have used a dihaploid line (2*n* = 2*X* = 18) derived from a lowland tetraploid individual through gynogenesis [36]. The term “dihaploid” is used to indicate the polyploid origin of the line and the homoeologous constitution of its subgenomes. Here we establish a standard reference karyotype that identifies visible features of individual switchgrass chromosomes and allows their unique discrimination. We demonstrate the presence of both additive and non-additive loci associated with rDNA and centromeric repeats, indicating subgenome divergence. We also document karyotype differences between upland and lowland genotypes.

## Results

### Chromosome measurement data

**Figure 1:**
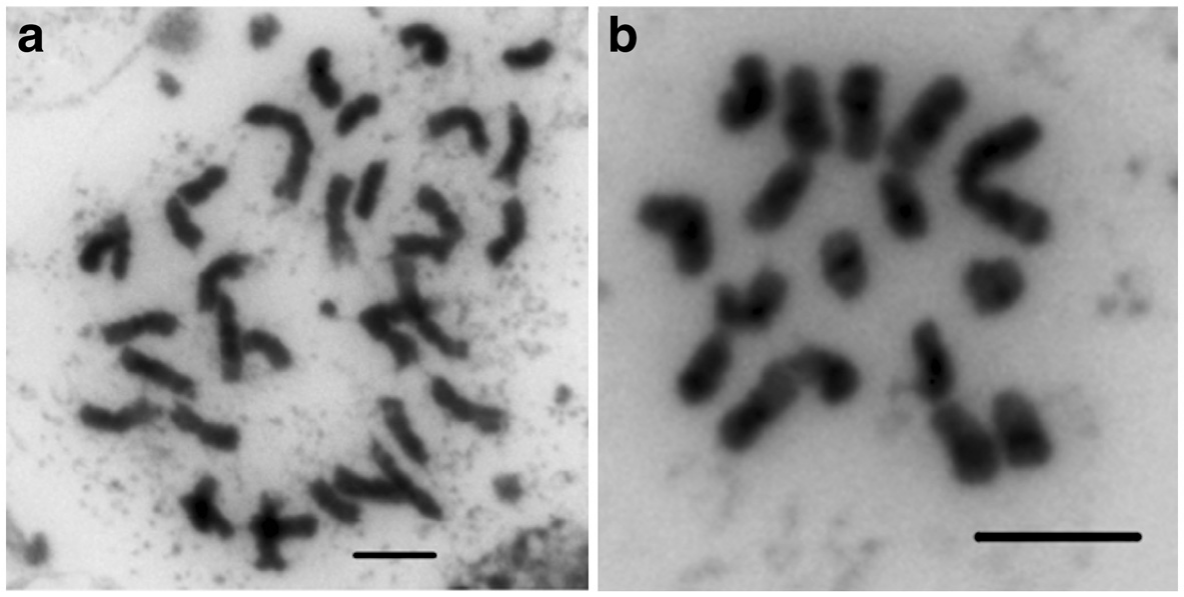
**Genome reduction in dihaploid individuals.** Chromosome squashes prepared from root tip cells confirmed 2*n* = 36 chromosomes in the tetraploid cultivar Kanlow (**a**) and 2*n* = 18 in dihaploid ALB280 (**b**). Scale bars = 5 μm.

**Figure 2:**
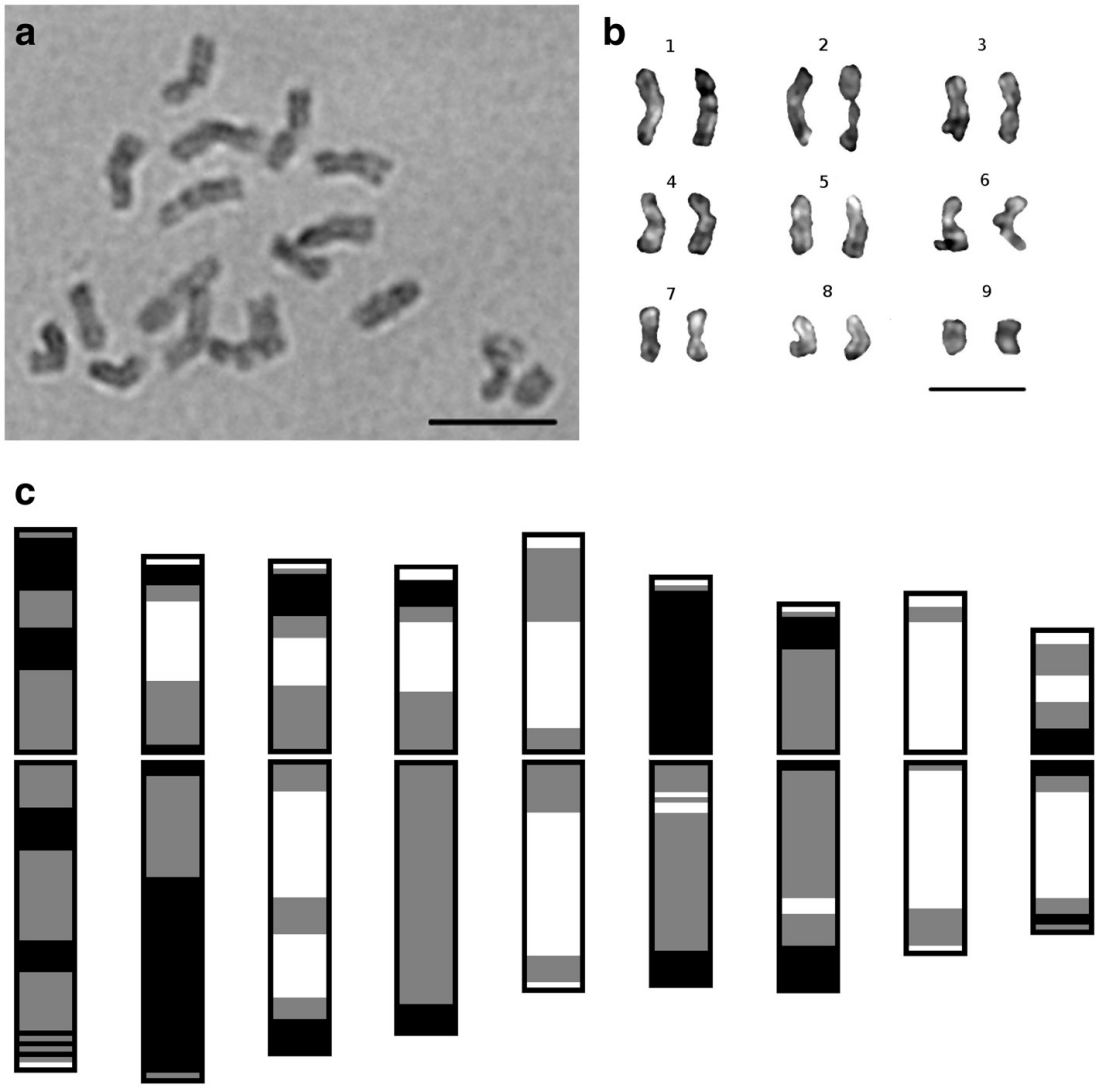
**Switchgrass Karyogram and Condensation Pattern (CP).** Mitotic prometaphase chromosome spreads were stained with acetocarmine, resulting in uneven condensation patterns (**a**). Homoeologous chromosomes were paired based on length, arm ratio, and CP data to develop a karyogram. The basic chromosome number of *n* = 9 is indicated (**b**). CP measurements were averaged for each chromosome across 10 mitotic spread images, resulting in a quantitative ideogram (**c**). Dark regions indicate highly-condensed heterochromatic areas, gray indicates intermediate condensation, and white indicates euchromatic areas. Scale bars = 5 μm.

Somatic chromosome counts made from root tip squash preparations confirmed the presence of 2*n* = 36 metaphase chromosomes in the tetraploid cultivar Kanlow (Figure 1a), which agrees with published data [14,41]. Chromosome numbers for ALB280 were shown to be 2*n* = 18 based on data from more than 50 metaphase plate preparations (Figure 1b). Image analyses of metaphase spreads from ALB280 (Figure 1b) were performed to produce the switchgrass karyotype. Digital measurements allowed for homoeologous chromosomes to be paired together and the base number *n* = 9 to be presented in a karyogram, from longest to shortest (Figure 2a,b). Morphological data from ten complete and undistorted chromosome spreads are presented in Table 1, indicating that switchgrass chromosomes are small to medium in length, ranging from 2.05 ± 0.16 μm to 4.10 ± 0.25 μm. Identification of the centromeres via FISH analyses (data presented below) allowed for measurement of chromosome arms and calculation of arm ratios (*r*). All chromosomes were classified as metacentric (m) based on parameters described by Levan et al. [42].

**Table 1:** **Morphology of*P. virgatum*chromosomes (ALB280)**

**Chromosome**	**Length**^**a**^**(μm ± SE)**	**Arm Ratio**^**b**^**(*r* ± SE)**	**Centricity Class**
1	4.10 ± 0.25	1.30 ± 0.09	m
2	3.73 ± 0.30	1.31 ± 0.06	m
3	3.38 ± 0.18	1.15 ± 0.07	m
4	3.26 ± 0.16	1.28 ± 0.13	m
5	3.05 ± 0.18	1.22 ± 0.16	m
6	2.82 ± 0.16	1.21 ± 0.06	m
7	2.61 ± 0.17	1.30 ± 0.08	m
8	2.35 ± 0.11	1.18 ± 0.06	m
9	2.05 ± 0.16	1.21 ± 0.04	m

^a^Average value of 10 chromosome pairs (μm).

^b^Arm ratio r (long/short) according to Levan et al. [42].

*SE* = standard error; *m* = metacentric.

### Condensation pattern

Chromosomes exhibit uneven staining due to variation in condensation pattern (CP) along their length. This distinctive CP, especially at the prometaphase stage of mitosis, was utilized as a diagnostic measurement for chromosome identification. CHIAS IV software [43] takes advantage of this uneven staining pattern and was used to generate a distinctive CP profile for each of the nine switchgrass chromosomes (Figure 2c). The same ten chromosome spread images used to generate the morphological data in Table 1 were analyzed with CHIAS IV software. Homoeologous chromosomes were paired in each prometaphase spread and then averaged together with homologs across all ten spreads. The resulting ideogram based on chromosome length and CP is presented in Figure 2c. Gray scale images allowed for distinct characterization of each switchgrass chromosome based on features such as the large, condensed region on the long arm of Chromosome 2, interstitial constrictions on both arms of Chromosome 1, and the large white areas indicating euchromatic regions of Chromosomes 5 and 8 (Figure 2c). Taken together with the length and arm ratio data, CP profiles allow each chromosome to be distinguished from all others in the karyotype.

### Localization of centromeres

**Figure 3:**
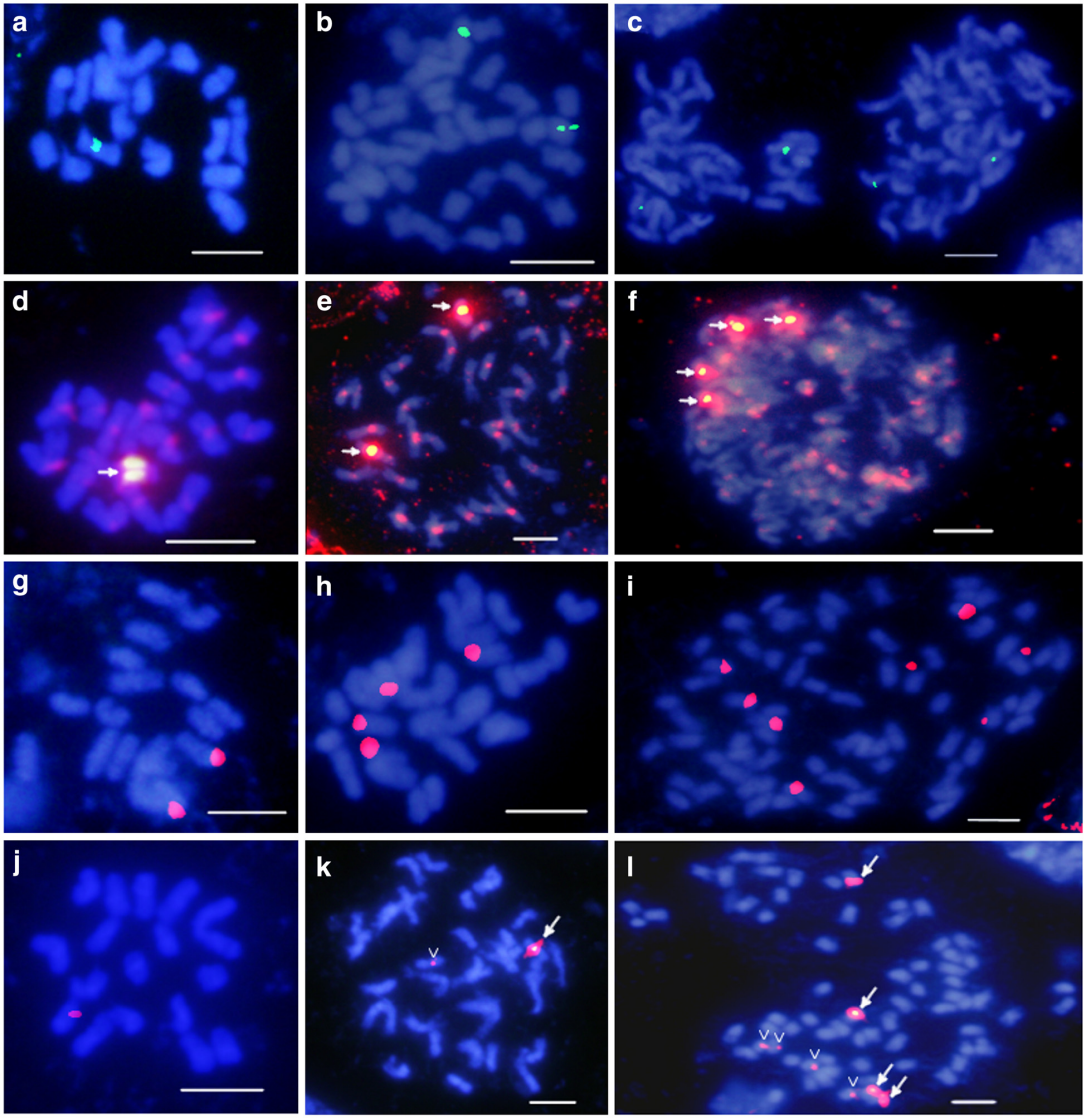
**FISH analyses of switchgrass chromosomes.** Comparative FISH analysis of switchgrass chromosomes was conducted using four different probe sequences: CentC (**a,b,c**); PviCentC (**d,e,f**); 45S rDNA (**g,h,i**); 5S rDNA (**j,k,l**). Three different switchgrass cytotypes were analyzed for each FISH probe: dihaploid 2*n* = 2*X* = 18 (**a,d,g,j**); tetraploid 2*n* = 4*X* = 36 (**b,e,h,k**); octoploid 2*n* = 8*X* = 72 (**c,f,i,l**). CentC signals (**a,b,c**) are indicated in *green*, whereas all other probes are labeled with *red* fluorescence. White arrows in **d**, **e**, and **f** indicate the strong PviCentC signal present on Chromosome 3 of the basic karyotype (*n* = 9). White arrows in **k** and **l** indicate “strong” 5S rDNA signals as compared to the “weak” 5S rDNA signals in each image (open arrowheads). Scale bars = 5 μm.

**Figure 4:**
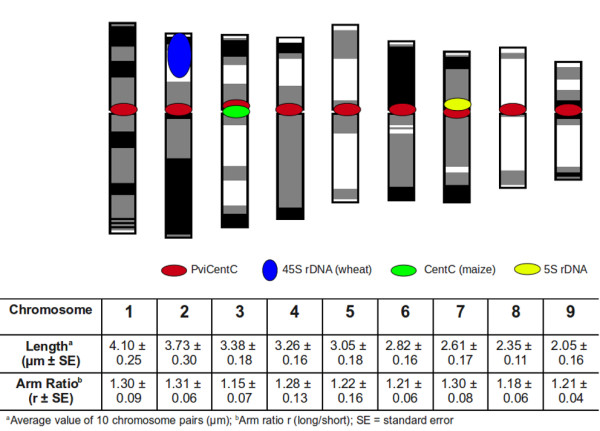
**Quantitative karyotype of switchgrass chromosomes.** An ideogram of the base *n* = 9 switchgrass chromosomes is displayed with corresponding FISH signal probes. Length and arm ratio measurements are averaged across 10 chromosome pairs.

A plasmid probe developed from the maize centromere repeat sequence CentC [44] (GenBank AF078922.1) was used for FISH of switchgrass chromosomes. Only one CentC signal was observed in mitotic chromosome spreads of dihaploid ALB280 (2*n* = 2*X* = 18) (Figure 3a). Upon FISH analysis of the lowland tetraploid cultivar Alamo (2*n* = 4*X* = 36), two CentC signals were evident (Figure 3b). Moreover, four CentC signals were present in chromosome spreads of the upland octoploid cultivar Grenville (2*n* = 8*X* = 72) (Figure 3c). Chromosome length and arm ratio data of ALB280 were used to determine that the CentC probe hybridizes to Chromosome 3 of the basic karyotype (Figure 4).

BLAST analysis of switchgrass 454 sequence data using the maize CentC and rice CentO sequences resulted in a subset of potential centromere-specific repeat sequences found in the switchgrass genome that, on average, were 73% identical to maize CentC (AF078922.1). Primers designed from the corresponding consensus sequence were used to amplify and label a switchgrass-specific centromere repeat sequence for FISH analysis. The labeled switchgrass centromere probe (PviCentC) produced a fluorescent signal on all 18 chromosomes of ALB280 (Figure 3d). In addition, one chromosome of ALB280 demonstrated a centromeric FISH signal significantly brighter than all the others. This PviCentC signal pattern was also present in all tetraploid cytotypes tested, where all centromeres were labeled and two chromosomes demonstrated a very high level of fluorescence (Figure 3e, cv. Kanlow). Octoploid cytotypes maintained this pattern, demonstrating four strong FISH signals on specific chromosomes while all others contained equal intensity signals at their centromeres (Figure 3f, cv. Cave-in-Rock). Length, arm ratio, and condensation pattern analyses identified the high fluorescing chromosomes as Chromosome 3, which is consistent with the maize CentC probe from which PviCentC was derived.

### 45S rDNA localization

The distribution of 45S rDNA on mitotic metaphase chromosomes of switchgrass was detected with FISH using the wheat pTa71 plasmid probe [45]. These results demonstrate that one pair of 45S rDNA signal was detected in chromosome spreads of ALB280, suggesting that the basic karyotype (n = 9) has one chromosome containing the 45S rDNA sequence (Figure 3g). When the tetraploid, lowland individual AP13 is probed, two pairs of 45S rDNA signals are present (Figure 3h). This pattern of one 45S rDNA signal per base genome (n = 9) also persisted in an individual from the octoploid upland cultivar Caddo, where eight 45S rDNA signals are visible (Figure 3i). Analysis of the morphology and FISH staining patterns in multiple spreads indicates that the 45S rDNA signal is found near the terminal end of the short arm of Chromosome 2 (Figure 4).

### 5S rDNA localization

Sequence data from 454 reads of dihaploid switchgrass ALB280 were used to BLAST against 5S rDNA sequences of several grass species, including sorghum, rice, maize, and wheat. The resulting sequence hits were used to amplify and fluorescently label a 314-basepair orthologous 5S rDNA sequence in switchgrass. These 5S rDNA FISH probes hybridized to a single chromosome of dihaploid ALB280 and to two chromosomes of the lowland tetraploid cultivar Kanlow (Figure 3j & k, respectively). Quantitative measurement data of ALB280 chromosomes localize the 5S rDNA signal to an interstitial region on Chromosome 7 (Figure 4). In the FISH image of Kanlow chromosomes, one 5S rDNA signal is strong and one 5S rDNA signal is weak (Figure 3k). In upland octoploid ecotypes, FISH signals for 5S rDNA demonstrate a different pattern than what would be predicted based on dihaploid and tetraploid data. Instead of two strong and two weak signals, the upland octoploid cultivar Caddo shows four strong and four weak hybridization sites (Figure 3l). This suggests a unique difference between upland and lowland ecotypes at the 5S rDNA locus.

### Ecotype variation at the 5S rDNA locus

**Figure 5:**
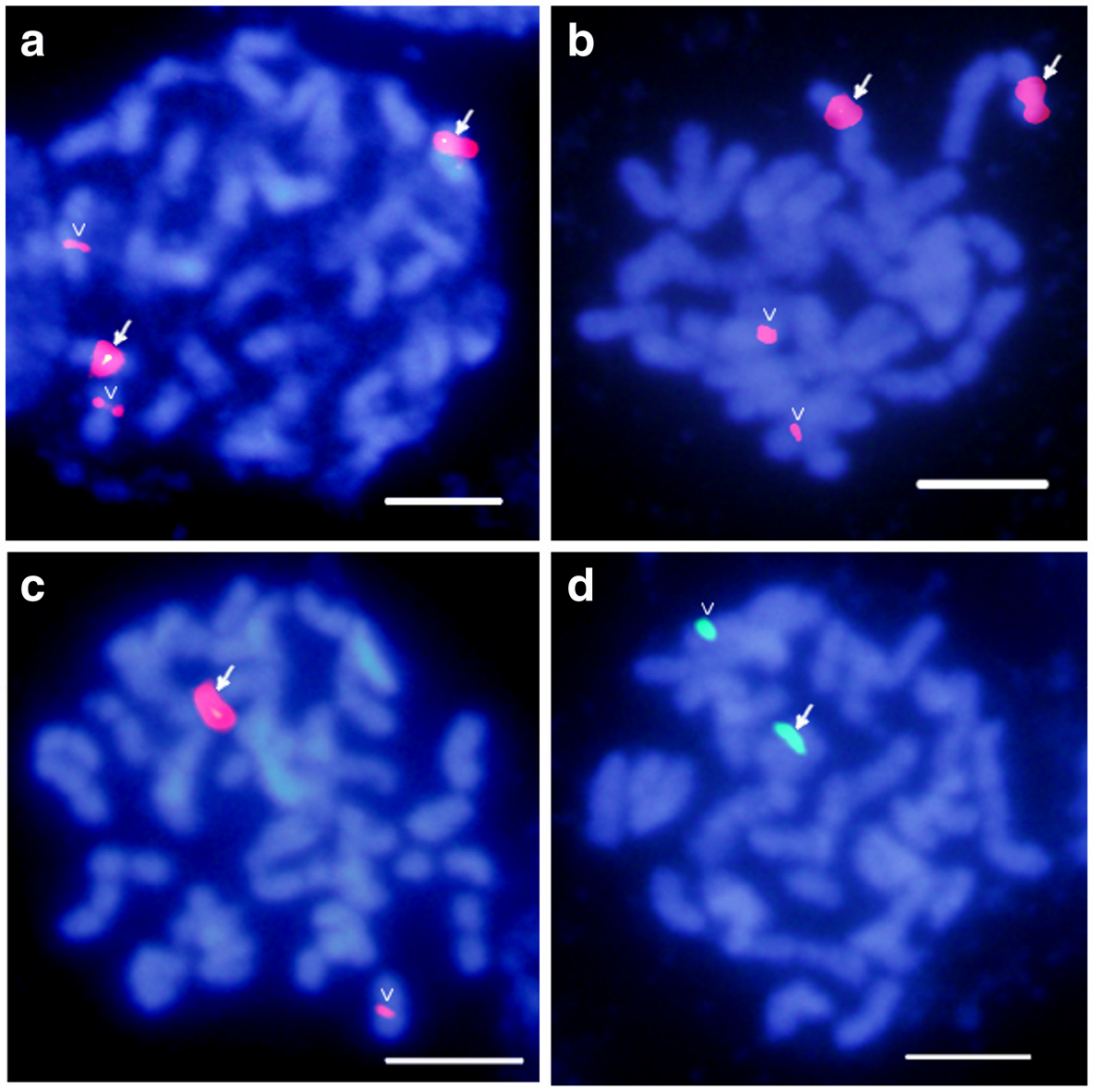
**Ecotype variation at the 5S rDNA locus.** FISH analyses of the 5S rDNA probe indicated signal variation between upland and lowland ecotypes. The upland tetraploid cultivars *Dacotah* (**a**) and *Summer* (**b**) were compared to the lowland tetraploid cultivars *Kanlow* (**c**) and *Alamo* (**d**). All FISH signals are of 5S rDNA, but both red and green fluorescence were used. White arrows in each image indicate “strong” signal patterns in comparison to “weak” signals in the same image (open arrowheads). The double “snake-eye” signal found in panel a simply indicates labeling of individual chromatids on the same chromosome. Scale bars = 5 μm.

To further examine differences between ecotypes of switchgrass at the 5S rDNA locus, upland tetraploid cultivars Dacotah and Summer were analyzed via FISH and compared to the lowland tetraploids Kanlow and Alamo. Two strong 5S rDNA signals and two weak signals (Figure 5) were observed in the upland tetraploids, which is consistent with the pattern seen in upland octoploids of four strong and four weak 5S rDNA signals (Figure 3l). In contrast, lowland tetraploids Kanlow and Alamo contain only one strong and one weak 5S rDNA signal (Figure 5). FISH data also support the conclusion that the dihaploid ALB280 has maintained one of the 5S rDNA loci from its tetraploid progenitor [36].

## Discussion

An accurate karyotype can incorporate physical measurements like total length and arm length ratios, but can also include landmarks such as heterochromatic knobs [46], patterns of chromatin condensation [43], and molecular features visualized by FISH [47]. Chromosome identification is critical for cytological analyses, as well as subsequent studies in genomics, taxonomy, and the evolution of polyploidy, enabling an understanding of the relationship between visible landmarks and genetic or physical map features [48]. To that end, the construction of a basic karyotype for switchgrass promises to facilitate genomic analyses. The somatic metaphase chromosomes of switchgrass are small, which may have limited examination of cytological features in earlier studies [11,13,49]. With the use of sophisticated imaging and molecular techniques, we are now able to present the first comprehensive karyotype for switchgrass that quantitatively distinguishes each of the nine base chromosomes of this bioenergy crop.

Use of a dihaploid line of switchgrass (ALB280) significantly simplified the karyotyping process. Acetocarmine- and DAPI-stained chromosome spreads allowed for visual pairing of homoeologous chromosomes in ALB280 and produced a karyotype based on total and relative lengths as well as arm ratios. In our experiments, a single switchgrass root tip preparation yielded an average of 20 or more dividing cells (prophase to metaphase). Chromosome spreads often resulted in a high frequency of nuclei at the pro-metaphase stage of mitosis. Pro-metaphase chromosomes demonstrated a characteristic condensation pattern (CP) along their length, corresponding to the compactness of the chromatids, which was used to create a quantitative idiogram [50]. This approach has also been useful in cytological analysis of *Brassica* species, sugarcane, and rice [51-53]. In conjunction with physical measurement data, CP data allowed us to unambiguously identify the small metacentric chromosomes of switchgrass.

Although morphological and CP data suggest a balanced karyotype in the dihaploid line ALB280 (2*n* = 2*X* = 18), FISH data presented here indicate that the subgenomes have different repetitive DNA content at PviCentC and 5S rDNA loci. This finding is in general agreement with the highly differentiated genomes indicated by linkage map data in which tetraploid ecotypes demonstrate fewer than expected markers mapping across subgenomes, and complete or near complete disomic inheritance [15]. It also agrees with the observation of 18 non-pairing univalents at diakinesis of meiosis in the dihaploid line [36]. FISH signal data at these genetic loci may point toward allopolyploid evolution of the switchgrass genome. However, these data are also consistent with natural loss of gene content following a whole genome duplication within a single species (autopolyploidy). To gain a greater understanding of the origins of switchgrass polyploidy, further phylogenetic analyses of the *Panicum* (s.s.) subgenus and/or genomic in situ hybridization (GISH) techniques should be used [1,27,54-56].

Under a simple additive model, FISH signals for the 5S rDNA locus and the CentC locus would be expected to be present in 2, 4, and 8 copies in the dihaploid, tetraploid, and octoploid lines, respectively. Surprisingly, only one FISH signal for these loci was observed in the dihaploid ALB280. In addition, the switchgrass-specific centromere probe, PviCentC, hybridized to all chromosomes of both subgenomes, but demonstrated a stronger FISH signal on Chromosome 3 of a single subgenome (same locus as maize CentC probe). This discrepancy in signal strength, particularly for the universal centromere probe PviCentC, suggests that a greater copy number of this repeat is present in Chromosome 3 of one subgenome than in all other chromosomes. Alternatively, or in conjunction, homology to the PviCentC FISH probe sequence may be much higher in Chromosome 3 than in all other labeled centromeres. The variation we observe at rDNA and centromeric loci may also be a result of the allogamous habit and self-incompatibility of switchgrass [20]. In outbreeding species of the genus *Secale*, high levels of repeat DNA polymorphism between homologous chromosomes have been documented [57]. Other outbreeding species in the *Lolium* and *Lolium-Festuca* complex demonstrate variation at rDNA loci [58,59], suggesting that hemizygosity in switchgrass may result from out-crossing. Also contributing to non-additive FISH signal data may be the high frequency of switchgrass aneuploids, particularly among octoploid cytotypes [18,40], which can lead to large-scale genetic changes and parental genome imbalance.

In our analyses of 45S rDNA loci, pairs of telomeric FISH signals demonstrated a regular, additive pattern up the ploidy series. Among tetraploid cultivars, our data are consistent with those of Costich et al. [18] in which all tetraploids analyzed (upland and lowland) demonstrated two pairs of telomeric 45S rDNA signals. However, in octoploid cultivars, Costich et al. [18] describe a large amount of variation in size, number, and location of 45S rDNA signals. The variation in 45S rDNA signal intensity seen in our analysis of the octoploid cv. Caddo (see Figure 3i) may suggest rDNA repeat variation and/or differences in probe hybridization affinity. With only a single upland octoploid (cv. Caddo) analyzed with 45S rDNA in this study, our results likely demonstrate one of many chromosomal constitutions for switchgrass octoploids. Overall, FISH analyses of tetraploid and octoploid individuals support elimination of rDNA and centromere sequences and demonstrates that patterns of subgenome differentiation are broadly maintained.

Our data also demonstrate unique ecotype differences at 5S rDNA loci. Variations in FISH signal patterns between upland and lowland tetraploids (and between lowland tetraploids and upland octoploids) provide features that distinguish these taxonomic divisions within switchgrass. We hypothesize that these variations in rDNA loci are related to both the phenotypic and geographic distribution differences observed between switchgrass ecotypes. In *Oryza* species, polymorphisms in the number, the chromosomal location, and the repeat length of rDNA loci have revealed species-specific and subgenome-specific FISH patterns [60]. The authors suggest that specific inversions, rDNA amplification, and locus transposition may have occurred during the process of *Oryza* evolution. Such a scenario may also be true for the divergence of switchgrass upland and lowland ecotypes. Classified cultivars of switchgrass are barely removed from the wild, and rapidly evolving rDNA loci are likely still undergoing change. The additive pattern of 5S rDNA loci seen between lowland tetraploids (2 signals) and upland tetraploids (4 signals) may be indicative of chromosomal rearrangements and rDNA changes that ultimately result in habitat adaptability differences between the two ecotypes. In addition, the conserved doubling pattern of 5S rDNA loci from upland tetraploid to upland octoploid further supports the maintenance of ecotype divergence across different cytotypes.

The presence of gene flow between switchgrass ecotypes and/or cytotypes has ramifications on the development and use of specific gene pools for switchgrass improvement. Therefore, knowledge of chromosome architecture and ploidy relationships is critical for cultivar development. Cytogenetic data presented here can be used to classify switchgrass plants according to ecotype and ultimately aid in identifying and isolating regionally adapted cultivars [61,62]. Switchgrass improvement through trait identification and breeding for significant heterotic effects also warrants the maintenance of independent gene pools [63,64]. Recent analyses of several putative upland and lowland accessions of switchgrass have identified the presence of natural hybrids between ecotypes as well as evidence of gene flow [22,23]. Results demonstrated a mixture of both cytotypes and phenotypes within hybrid populations, suggesting a long history of gene flow. In addition, genetic marker data indicated that gene flow is bidirectional, from upland to lowland and from lowland to upland [23]. A quantitative understanding of whole switchgrass chromosomes will help in distinguishing hybrid genotypes and aid in tracking genome sources during directed breeding programs. The use of FISH and GISH can identify translocations and/or introgression of new chromosome sources [25,65], but can also be used to identify ancestral genomes that contribute to the evolution of polyploid species such as switchgrass [27,66].

The development of a species karyotype, with unambiguous identification of individual chromosomes, is also critical for the integration of genetic and physical map data. Genetic maps of switchgrass have been constructed using SSR and STS markers [15]. Hybridization of these genetically mapped markers to switchgrass chromosomes would lead to definitive assignment of linkage groups. Fluorescence detection of single- and low-copy sequences through the use of BAC clones as probes has proven highly successful in many species, including rice [67], maize [68], and sorghum [69]. BAC libraries for switchgrass have recently been developed and may be utilized for the integration of linkage and physical maps through BAC-FISH probing. BAC sequences would also be useful in flow sorting of chromosome fractions for physical gene mapping and construction of chromosome-specific libraries [70]. In this light, FISH technology will continue to be a valuable tool in understanding genome structure in switchgrass.

## Conclusions

The relatively small size and lack of distinguishing features among switchgrass chromosomes have likely deterred detailed karyotype analysis in the past [11,40]. Through the use of sophisticated molecular, cytological, and imaging techniques, we describe a quantitative karyotype for *P. virgatum* L. that distinguishes individual chromosomes. Our data support the genetic divergence of the two subgenomes within switchgrass and provide a foundation for studying the evolution of polyploidy in this bioenergy crop. We also demonstrate karyotype differences between upland and lowland ecotypes that will aid in identifying and maintaining diverse gene pools for future breeding strategies. Additional cytogenetic analyses of switchgrass chromosomes are necessary, but data presented here provide a quantitative foundation on which genomic and genetic studies can continue to advance. This karyotype will enable aneuploid stocks as well as alien substitution and addition lines to be characterized and used by others for classical genetic analysis and introduction of new variation.

## Methods

### Plant material and growth conditions

Dihaploid switchgrass plants (2*n* = 2*X* = 18) were identified from among the progeny of a biparental cross between two lowland tetraploid cultivars, Kanlow and Alamo, as previously described [36]. Tetraploid and octoploid cultivars used in cytological and FISH analyses were obtained either from colleagues in Lincoln, NE or from the National Genetic Resources Program (NGRP, Beltsville, MD). Cultivar accessions used in this study are described in Additional file 1 Table S1. Seeds of all ecotypes were stratified for 3 weeks at 4°C before germinating on wet filter paper and transplanting to the greenhouse. Plants were maintained in the greenhouse at 72°F, under supplemental lighting (16 h day), watered as needed, and fertilized weekly with general purpose 20-20-20 fertilizer.

### Mitotic chromosome preparation

Mitotic chromosome spreads were generated following a protocol by Zhang and Friebe [71] with a few modifications. Actively growing root tips were excised from greenhouse grown plants, pretreated in ice cold water for 18–24 h, and then fixed in 3:1 ratio of 95% ethanol and glacial acetic acid at 4°C, overnight. Root material was either used immediately for slide preparation or stored in fixative at −20°C for up to several months. For slide preparation, the root tips (0.5-1.0 cm) were washed twice for 5 min in 0.01 M citrate buffer and digested in an enzyme mixture of 50 mg/ml Onozuka R-10 cellulase and 30 mg/ml Macerozyme (Phytotechnology Labs, Shawnee Mission, KS) at 37°C. Digestion times varied from 30 mins to 2.5 h, depending on the thickness and degree of lignification in the root tip. Softened root tips were then washed for 5 min in 0.01 M citrate buffer and transferred to a slide. Forceps and a scalpel were used to carefully excise the white tissue just behind the root cap containing actively dividing mitotic cells. All other root tissues were removed and the remaining cells were macerated in a few drops of 1% acetocarmine stain. A coverslip was placed over the stained tissue and even pressure was applied to generate mitotic chromosome spreads. Slides were viewed under phase-contrast microscopy to identify spreads optimal for use in FISH analysis.

### Probe DNA labeling and fluorescence in situ hybridization (FISH)

The clone pTa71 from *Triticum aestivum* L. [45] as used to identify the 45S rDNA (18 S-5.8 S-25 S rRNA) gene sequence. The CentC probe sequence [44] was generously provided by Dr. Zac Cande as a plasmid clone derived from PCR products of *Zea mays* DNA. The 5S rDNA probe for switchgrass was designed using 5S rDNA sequences from maize, rice, and sorghum to identify similar sequences from a database of switchgrass 454 sequence reads from a cv. Alamo individual using BLASTN. Primers (FP 5'-AGCACGCTTACGTTCGAGTTCTGA-3'; RP 5'-AGAATGGCTAGATGCGCGGAGAAT-3') were developed from the resulting BLASTN hits with highest e-values. The PviCentC probe was amplified from switchgrass gDNA using PCR primers designed using 454 sequence reads that were identified as similar to maize CentC and rice CentO sequences using BLASTN [72]. Analysis of resulting BLAST hits was used to infer the PviCentC sequence from existing switchgrass raw genome sequence data. The resulting consensus sequence was used to design primers for the switchgrass-specific centromeric repeat probe (FP 5'-CATGCCCAATCCACTTCTTTAGGC-3'; RP 5'-CAACTTACGGGAAGCACAAAGTGG-3'). The resulting 143-bp PCR product was labeled with either digoxigenin-11-dUTP or biotin-16-dUTP using nick translation or PCR (Nick Translation Kit; PCR DIG Probe Synthesis Kit, Roche Applied Sciences, Indianapolis, IN), in accordance with manufacturer instructions. Hybridization and post-hybridization wash procedures were performed as previously described [73]. Chromosomes were counter-stained with 4',6-diamidino-2-phenylindole (DAPI).

### Microscopy and image analysis

Digital images were recorded using an Olympus BX51 epifluorescence microscope (Olympus Corporation, Center Valley, PA) with a DP70 CCD (charge coupled device) camera and suitable monochrome filter sets (Chroma Technology, Rockingham, VT). Images were processed using GIMP 2.6 (GNU Image Manipulation Program) for Linux. Chromosome length measurements and arm ratios were determined manually using GIMP 2.6 and confirmed with automated measurements in CHIAS IV software (Chromosome Image Analyzing System IV) [43]. Analysis of chromosome condensation patterns were performed by the CHIAS IV system using the macro programs written by Seiji Kato, Nobuko Ohmido, and Kiichi Fukui. Analysis of FISH data was conducted in GIMP 2.6 by overlaying a probe signal image on top of the corresponding DAPI stain image. Adjustments were made to the transparency of the top (FISH signal) layer to demonstrate signal and chromosome alignment.

### Karyotype analysis

Karyotype analyses were performed on ten undistorted and non-overlapping chromosome spreads from the dihaploid ecotype ALB280 (2*n* = 2*X* = 18). Homoeologous chromosomes from acetocarmine and/or DAPI stained spreads were paired based on total length, arm ratio, and condensation pattern. Physical length measurements were taken for each chromosome using GIMP 2.6 and CHIAS IV imaging software and by converting pixel lengths into microns. Statistical *t* tests were conducted to confirm that homoeologous pairs were not significantly different from one another. Chromosome arms were measured from the centromere to the tip of each arm and the centricity class was determined from arm ratio (r = long-arm length/short-arm length), as previously described [42]. Whenever possible, FISH signal data were used to pair homoeologous chromosomes and measurement data were averaged for each chromosome in the karyotype across all ten mitotic spreads. Chromosomes were numbered 1–9 from the longest to the shortest and paired with FISH signal data to define the karyotype (*n* = 9). In addition, condensation patterns (CP) of all ten mitotic spreads were subjected to analysis with CHIAS IV software to aid in distinguishing chromosomes.

### Dihaploid lines of switchgrass

Karyotype analysis of the base 9 chromosomes of switchgrass was significantly aided by the use of a dihaploid individual (2n = 2X = 18) previously identified from among the progeny of a controlled cross between two tetraploid cultivars (2n = 4X = 36), Kanlow and Alamo [36]. This dihaploid line (ALB280) was initially distinguished from parental lines by its reduced heterozygosity and was subsequently confirmed through estimation of C values by flow cytometry. This analysis indicated that ALB280 had a 2 C value of 1.48, approximately half that of a tetraploid F1 reference individual (ALB881) and the Alamo male parent (ALBA4) which had 2 C values of 2.61 and 2.75 pg, respectively [36].

## Competing interests

The authors declare no competing interests of any kind.

## Authors’ contributions

CT, GS, and HY conceived and designed the study. GS and CT provided critical plant and molecular materials and conducted sequence analysis for the design of FISH molecular probes. HY performed the experiments and analyzed the data. HY authored the manuscript with critical help from CT and GS. All authors read and approved the final manuscript.

## Supplementary Material

Additional file 1 **Table S1.**Switchgrass (*Panicum virgatum* L.) cultivars included in this study [74]. Click here for file
