# Supplementary material for: Karyotype variation is indicative of subgenomic and ecotypic differentiation in switchgrass
Source: BMC Plant Biol. 2012 Jul 26;12:117. doi: 10.1186/1471-2229-12-117 (PMC3492167; doi:10.1186/1471-2229-12-117)
Supplement: Additional file 1 — Table S1.Switchgrass (Panicum virgatum L.) cultivars included in this study [74]. [file 1471-2229-12-117-S1.doc]

Supplemental Table S1 Switchgrass (Panicum virgatum L.) cultivars included in this study

| **Cultivar** | **Accession**  **Number** | **Ecotype** | **Ploidy** | **Origin^a^** |
| --- | --- | --- | --- | --- |
| ALB280 | Grif 17268 | Lowland | 2x | Albany, CA |
| Alamo | PI 422006 | Lowland | 4x | Texas |
| Kanlow | PI 421521 | Lowland | 4x | Oklahoma |
| Dacotah | PI 537588 | Upland | 4x | North Dakota |
| Summer | PI 642191 | Upland | 4x | Nebraska |
| Caddo | PI 476297 | Upland | 8x | Oklahoma |
| Cave-in-Rock | PI 469228 | Upland | 8x | Illinois |
| Grenville | PI 414066 | Upland | 8x | New Mexico |

^a^ Origin as defined by Alderson et al. (1995) [74]
